# Vascular Endothelial Growth Factor A and VEGFR-1 Change during Preimplantation in Heifers

**DOI:** 10.3390/ijms21020544

**Published:** 2020-01-15

**Authors:** Daniel Chiumia, Anna-Katharina Hankele, Anna E. Groebner, Katy Schulke, Horst-Dieter Reichenbach, Katrin Giller, Valeri Zakhartchenko, Stefan Bauersachs, Susanne E. Ulbrich

**Affiliations:** 1 ETH Zurich, Animal Physiology, Institute of Agricultural Sciences, 8092 Zurich, Switzerland; daniel.chiumia@usys.ethz.ch (D.C.); anna-katharina.hankele@usys.ethz.ch (A.-K.H.); 2 Physiology Weihenstephan, Technical University of Munich, 85354 Freising, Germany; annaehennersperger@gmail.com (A.E.G.); katyschulke@yahoo.de (K.S.); 3 Bavarian State Research Center for Agriculture, Institute of Animal Breeding, 85586 Poing, Grub, Germany; Horst-Dieter.Reichenbach@lfl.bayern.de; 4 ETH Zurich, Animal Nutrition, Institute of Agricultural Sciences, 8092 Zurich, Switzerland; katrin.giller@usys.ethz.ch; 5 Chair for Molecular Animal Breeding and Biotechnology, Gene Center, Ludwig-Maximilians University, 81377 Munich, Germany; V.Zakhartchenko@gen.vetmed.uni-muenchen.de (V.Z.); stefan.bauersachs@uzh.ch (S.B.); 6 Vetsuisse Faculty Zurich, University of Zurich, Eschikon 27, AgroVet-Strickhof, 8315 Lindau (ZH), Switzerland

**Keywords:** angiogenesis, bovine, conceptus, endometrium, IVF, SCNT

## Abstract

Vascular endothelial growth factor A (VEGFA) plays a critical angiogenic role in the endometrium of placentalia during preimplantation. The role of VEGFA and its receptors is not fully characterised in bovine reproduction. We analysed the mRNA expression of VEGFA isoforms 121, 165 and 189, and VEGF receptors 1 and 2 in three experimental settings (A, B and C). We compared intercaruncular endometrium of cyclic to pregnant heifers at Days 12, 15 and 18 post insemination (Day 0), and between Day 15 and Day 18 conceptuses (A). We further compared caruncular versus intercaruncular endometrium at Day 15 (B), and endometrium of heifers carrying embryos originating from somatic cell nuclear transfer (SCNT) versus in vitro fertilisation (IVF) at Day 18 (C). Endometrial VEGFA protein was localised and quantified. Pregnant heifers displayed lower intercaruncular endometrial mRNA expression of VEGFA-121 (*p* = 0.045) and VEGFA-189 (*p* = 0.009) as well as lower VEGFA protein abundance (*p* < 0.001) at Day 15. The VEGFA protein was localised in intercaruncular luminal, glandular epithelium and in tunica muscularis of blood vessels. At Day 15, caruncular endometrium displayed higher VEGFA mRNA expression than intercaruncular endometrium (*p* < 0.05). Intercaruncular endometrial VEGFA protein at Day 18 was higher in abundance in SCNT than in IVF (*p* = 0.038). Therefore, during preimplantation in cattle, there may be a need for timely physiological reduction in intercaruncular endometrial VEGFA expression in favour of the caruncular area to facilitate a gradient towards the implantation sites. A higher expression of VEGFA in SCNT may predispose for later placentation abnormalities frequently observed following SCNT.

## 1. Introduction

Early embryonic losses heavily affect bovine reproduction and contribute to low calving rates (50 to 60%) despite high fertilisation rates (~90%) [1]. During the preimplantation period when the uterus undergoes vascular changes to create an optimal environment for the rapidly growing demands of the conceptus, vascular endothelial growth factor A (VEGFA) is a critical angiogenic factor in both humans and animals [2]. The dimeric glycoprotein VEGFA is a member of the cysteine-knot growth factor family that include VEGFB, VEGFE, VEGFC, VEGFD and placental growth factor. The VEGFA is by far the most known potent and pivotal factor for angiogenesis [3,4]. Because of alternative mRNA splicing of the exons from the VEGFA gene, different VEGFA isoforms are formed. The most abundant isoforms are VEGFA-121, VEGFA-165 and VEGFA-189 [5]. The isoforms differ first in their heparin and heparan sulfate-binding abilities [4] and second regarding whether the isoform encodes a pro- or antiangiogenic protein [5]. Among these three isoforms, VEGFA-165 is the prototypical VEGFA isoform that induces a robust agonist through the receptor signaling responses and is taken as a reference isoform for investigating other VEGFA isoforms [6]. Relative to both VEGFA-121 and VEGFA-189, VEGFA-165 has a higher binding capacity and affinity to induce downstream angiogenic signaling [6,7,8]. The activities of VEGFA are mediated by the membrane receptors VEGFR-1 and VEGFR-2, which are the universal regulators of endothelial cell functions [9]. Precisely, VEGFR-1 regulates proliferation of endothelial cells during angiogenesis and VEGFR-2 is involved in migration and proliferation of endothelial cells during vessel maintenance [10].

In bovine reproduction, VEGFA substantially contributes to follicular development, functional maintenance of the corpus luteum, early development of the conceptus and establishment of the placentation processes [11,12,13]. Ortega Serrano et al. [14] observed that VEGFA contributes to the selection of the dominant follicle in dairy cows. Luo et al. [11] showed that 5 ng/mL of recombinant human VEGFA-165 added to the maturation medium increased both the cleavage rate of bovine oocytes and the conceptus developmental rate to the 8-cell stage. The recombinant VEGFA-165 treatment in the latter study had nevertheless no influence on subsequent developmental rates of the 8-cell stage bovine conceptuses on Days 6, 7 and 8 in vitro. Further, changes in colocalisation of VEGFA in the vasculature between the implantation stages (from Days 18 to 24) and later stages of gestation in bovine substantiate the necessity of angiogenesis during implantation [1]. Thus, the importance of VEGFA during folliculogenesis and early conceptus development until Day 8 and from the implantation stage (Days 18 to 24) onwards until term has been reported for cattle. The contribution of VEGFA beyond Day 8 of pregnancy and prior to implantation is yet to be understood. Inferring from the mouse model, it is possible to disrupt angiogenesis with a single dose of an angiogenesis inhibitor before or shortly after implantation, resulting in resorption of the conceptuses [2]. Furthermore, during periimplantation stages in mice, the VEGFA and VEGF receptors are expressed in the endometrium and facilitate vascular hyperpermeability, which is necessary for blastocyst implantation [2]. The intercaruncular and caruncular endometrium in bovine are both critical for the development of the conceptus, as they are the major sites for histotroph synthesis and placentome formation during pregnancy, respectively [15].

Despite recent advances in breeding and biotechnology fields [16,17], techniques such as somatic cell nuclear transfer (SCNT) still result in low number of live births in cows. Specifically, there are severe aberrations in vascular development during the early intrauterine development that result in high embryonic losses of cloned pregnancies [10,16]. Bauersachs et al. [16] as well as Mansouri et al. [18] suggested that associated placentation malfunctions in clone pregnancies emanate from abnormal interactions between the conceptus and the endometrium that develop during the preimplantation phase. Additionally, during second and third trimester, cows carrying SCNT are reported to have abnormally enlarged and less placental cotyledons, develop hydroallantois and have enlarged umbilical cords [16].

We aimed at unravelling if the expression of endometrial and embryonic VEGFA isoforms and the associated receptors are modulated during the preimplantation period in cattle. Furthermore, we tested the hypothesis that prior to implantation, the caruncular areas display a higher VEGFA abundance to prepare for implantation than the intercaruncular endometrium areas. We finally questioned if VEGFA was altered in heifers gestating cloned conceptuses.

## 2. Results

### 2.1. Study A: Cycling versus Pregnant Heifers

#### 2.1.1. The Concentration of P4 and E2 in the Serum of Pregnant and Nonpregnant Heifers

We observed a higher concentration of P4 in the serum of pregnant heifers than nonpregnant heifers at Days 15 and 18 (*p* < 0.05). There were no significant differences between the groups in P4 concentrations at Day 12 (Table 1).

High P4 in pregnant heifers at Days 15 and 18 was expected due to the continued secretion of P4 from the corpus luteum in pregnant cattle. Comparing the nonpregnant and pregnant heifers, we did not find any significant differences in E2 concentrations at Days 12, 15 and 18 post insemination.

#### 2.1.2. mRNA Expression of VEGFA Isoforms and VEGF Receptors in the Endometrial and Conceptus Tissues

**Figure 1:**
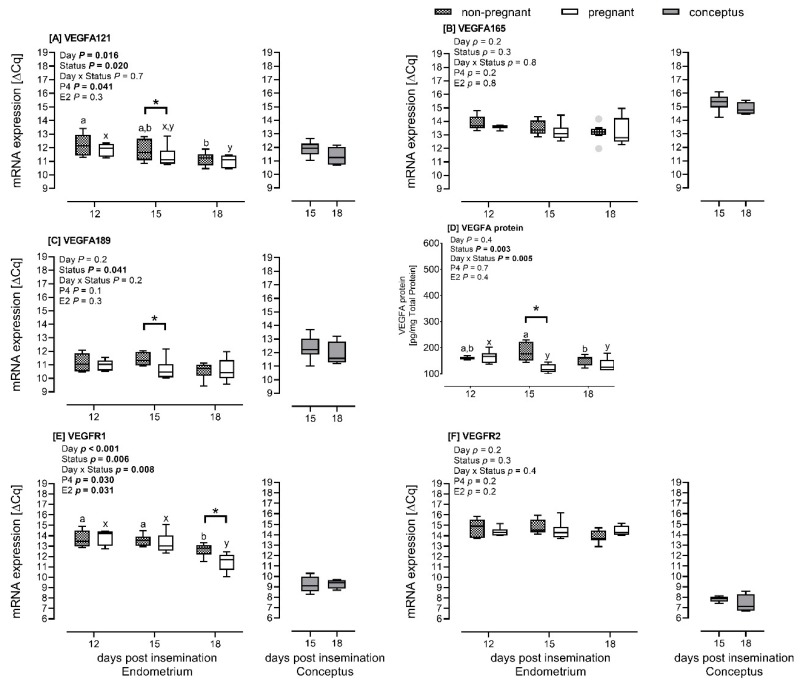
mRNA expression of vascular endothelial growth factor A (VEGFA) isoforms (**A**–**C**), VEGF receptors (**E**,**F**) and VEGFA protein abundance (**D**) in the intercaruncular endometrium of nonpregnant (*n* = 5 to 8) and pregnant (*n* = 5) Simmental heifers at Days 12, 15 and 18 post insemination as well as conceptuses at Days 15 (*n* = 8) and 18 (*n* = 4) (Insemination = Day 0). Asterisk (*) indicates significant differences between groups and letters significant differences within nonpregnant (a,b,c) and pregnant (x,y,z) groups. Abbreviations: P4 = progesterone and E2 = estradiol-17β. Results for mRNA expression presented as mean delta quantitative cycle (ΔCq) ± standard error of the mean (SEM). Differences with *p* < 0.05 were considered as significant.

The pregnancy status of the heifers affected the mRNA expression of endometrial VEGFA-121 (*p* = 0.045) and VEGFA-189 (*p* = 0.009) at Day 15 (Figure 1A,C) and VEGFR-1 receptor (*p* < 0.001) at Day 18 (Figure 1E).

The mRNA expression was lower in pregnant heifers than in nonpregnant heifers. The endometrial VEGFA-121 mRNA expression was significantly lower when comparing Day 12 to Day 18 (Figure 1A). At Day 15, the abundance of VEGFA-121 mRNA was the same as Days 12 and 18 post insemination. The changes in VEGFA-121 mRNA expression from Days 12 to 18 were similar within the respective pregnant and nonpregnant groups. For the VEGFR-1 mRNA expression, the transcript abundance was stable from Days 12 to 15, and decreased (*p* < 0.001) from Days 15 to 18 overall and within the pregnant and nonpregnant groups (Figure 1E). The concentration of P4 contributed to the changes in mRNA expression we observed for VEGFA-121 (*p* = 0.041) (Figure 1A). Both P4 and E2 concentrations affected the mRNA expression of the VEGFR-1, *p* < 0.05 (Figure 1E). The mRNA expression of endometrial VEGFA-165 and VEGFR-2 was the same according to status and day and was not affected by P4 and E2 concentrations (Figure 1B,F).

The conceptus mRNA expression did not differ between Days 15 and 18 for the VEGFA isoforms and VEGF receptors (*p* > 0.05). We further observed that at Day 15, the conceptus VEGFA mRNA expression in pregnant heifers was similar (VEGFA-121) or higher (VEGFA-165 and VEGFA-189) than the endometrial VEGFA mRNA expression (Figure 1A–C; folds: 2.1 and 1.7, respectively). The conceptus mRNA expression of the VEGF receptors was lower in conceptuses than in the endometrium (Figure 1E,F; folds: 4.1 and 7.1, respectively).

#### 2.1.3. Endometrial VEGFA Protein Abundance

We observed a difference in VEGFA protein abundance in the endometrium between pregnant and nonpregnant heifers at Day 15 (*p* < 0.001). The protein abundance was lower in pregnant than in nonpregnant heifers (Figure 1D), thus showing a similar pattern as observed for the mRNA expression. In pregnant heifers, the endometrial VEGFA protein abundance decreased (*p* = 0.004) from Days 12 to 15 and remained stable until Day 18. In the endometrium of nonpregnant heifers, VEGFA protein abundance did not differ between Days 12 and 15 but decreased between Days 15 and 18 (*p* = 0.014). 

#### 2.1.4. Localisation of VEGFA in the Endometrium

**Figure 2:**
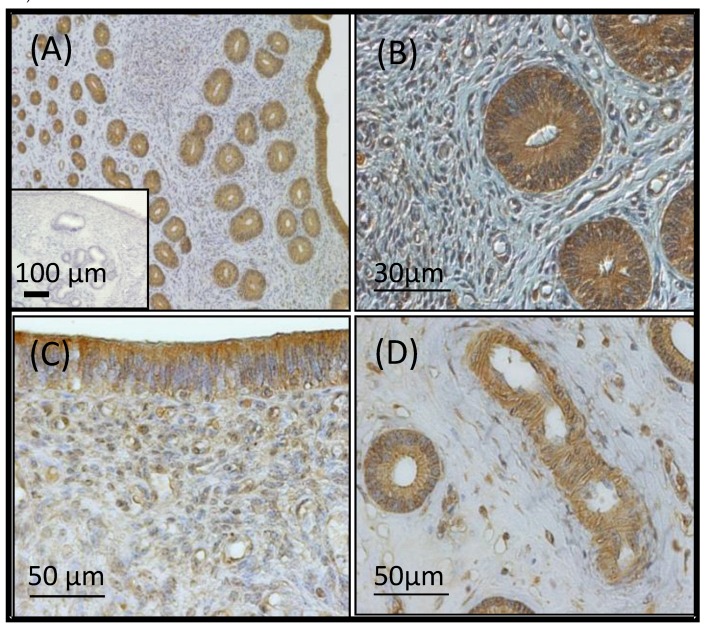
VEGFA immunohistochemistry: an overview of the endometrium (**A**) and close-up views of an endometrial gland (**B**), the luminal epithelium (**C**) and a blood vessel (**D**). Insert (**A**) displays the negative control where serum was used instead of the primary antibody. All cells stained positive for vascular endothelial growth factor A without differing in intensity over time between pregnant and nonpregnant heifers.

The VEGFA protein was expressed in the intercaruncular luminal and glandular epithelium without differing in intensity over time in pregnant and nonpregnant heifers. In addition, endothelial cells of endometrial capillaries and the tunica media of arterioles stained positive for VEGFA as well (Figure 2).

### 2.2. Study B: Caruncular versus Intercaruncular Endometrium in Pregnant Heifers 

**Figure 3:**
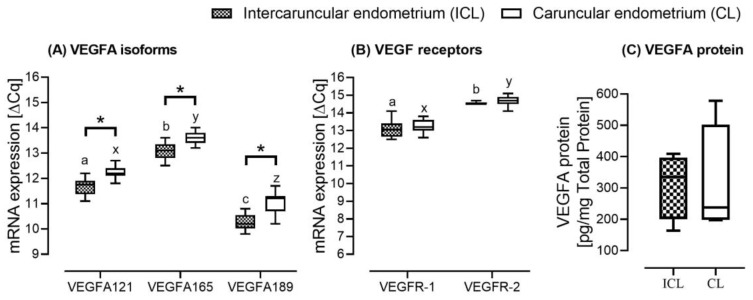
Day 15 post insemination vascular endothelial growth factor A (VEGFA) isoforms (**A**), VEGF receptors (**B**) mRNA expression and protein abundance (**C**) in the intercaruncular endometrial (ICL) and caruncular endometrial (CL) tissues in pregnant Angus heifers (*n* = 12). Asterisk (*) indicates significant differences between tissues (intercaruncular vs. caruncular) for the respective genes and letters significant differences in mRNA expression within the intercaruncular endometrium (a,b,c) and the caruncular endometrium (x,y,z) when comparing the genes. Significant differences *p* < 0.05.

We assessed the intercaruncular and caruncular endometrial tissues mRNA expression at Day 15 post insemination. The mRNA expression of all VEGFA isoforms at Day 15 in the caruncular endometrial tissue was higher than the mRNA expression in the intercaruncular endometrial tissue (*p* < 0.05) (Figure 3A).

The mRNA expression of VEGFA-165 was higher than the expression of the other two isoforms (*p* < 0.001). For the VEGF receptors, the mRNA expression did not differ between intercaruncular and caruncular endometrial tissues (Figure 3B). Day 15 post insemination total VEGFA protein abundance was not significantly different between the intercaruncular and caruncular endometrial tissues (Figure 3C). We only observed a numerical difference, with a higher protein abundance in the caruncular than in the intercaruncular endometrial tissues (477 ± 104 versus 720 ± 94).

### 2.3. Study C: SCNT versus IVF Pregnancies

**Figure 4:**
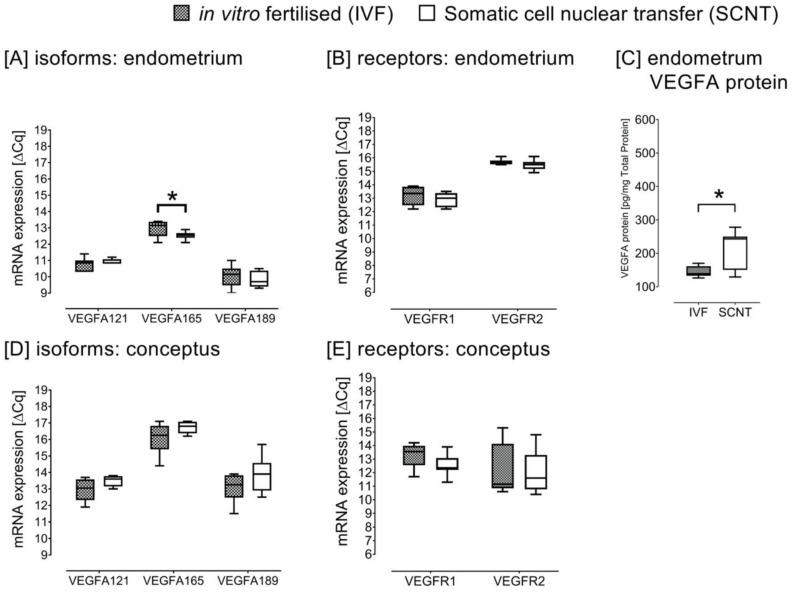
Day 18 post insemination vascular endothelial growth factor A (VEGFA) isoforms (**A**) and VEGF receptors (**B**) mRNA expression in the intercaruncular endometrium of heifers gestating the in vitro fertilised (IVF) and somatic cell nuclear transfer (SCNT) conceptuses. Endometrial VEGFA protein abundance (**C**) and conceptus mRNA expression (**D**,**E**) are presented (*n* = 8 for the respective IVF and SCNT treatments). Asterisk (*) indicates significant differences at *p* < 0.05.

Th gene expression abundance of the VEGFA-165 in the intercaruncular endometrium of heifers carrying SCNT-derived conceptuses was lower than in the respective endometrium of heifers with IVF-derived conceptuses at Day 18 (*p* = 0.045) (Figure 4A). There were no differences in the mRNA expression of the receptors in the endometrial tissues (Figure 4B). 

Contrarily, in the endometrium of heifers gestating SCNT-derived conceptuses, we observed a higher VEGFA protein abundance than in the endometrium of heifers gestating IVF-derived conceptuses (*p* = 0.038) (Figure 4C). The VEGFA protein abundance in the endometrium of the IVF group was comparable to Day 18 endometrial VEGFA protein abundance of nonpregnant and pregnant heifers in study A. The mRNA expression of the VEGFA isoforms and receptors in the conceptuses was not different between SCNT and IVF (Figure 4D,E). We further noted that the mRNA expression of VEGFA-165 was higher than of VEGFA-121 and VEGFA-189 in both the endometrium and the conceptuses. The latter observation was consistent in all the three experimental settings (A, B and C).

## 3. Discussion

We observed that the mRNA expression of the soluble VEGFA-121, membrane bound VEGFA-189 isoforms [19] and the total VEGFA protein abundance in the intercaruncular endometrium were significantly lower in pregnant than in nonpregnant heifers at Day 15 post insemination. Among other functions, both VEGFA isoforms promote endothelial cell growth and vascular permeability, and VEGFA-189 when compared to VEGFA-121 has low mitogenic activity probably due to its attachment to the extracellular matrix [5,20]. These results suggest that during the preimplantation period in bovine, there is spatiotemporal physiological reduction in the intercaruncular endometrial VEGFA, possibly to initiate the implantation process at the caruncular implantation sites. In pigs, implantation occurs at Day 13 post insemination. The porcine endometrial VEGFA mRNA expression was shown to be reduced from Days 9 to 12 and then gradually increased from Day 18 of pregnancy onwards, concurrent with a reduction in VEGF protein at Days 16 and 17 [21]. We noted that the endometrial VEGFA-121 and VEGFR-1 mRNA expression patterns we analysed in bovine were comparable to porcine endometrium mRNA expression during the preimplantation period [21]. Thus, freely diffusible VEGFA-121 may possibly play a critical role during the initiation process of implantation through VEGFR-1 receptor, as speculated earlier [1]. In this study, we observed that VEGFR-1, which regulates proliferation of endothelial cells during angiogenesis [10], was significantly reduced at Day 18 in the intercaruncular endometrium of the pregnant heifers. Ferrara et al. [22] indicated that a mediation function of VEGFR-1 in the vascular endothelium is to induce the release of tissue specific growth factors in a vascular bed-specific fashion. Thus, VEGFR-1 has the capacity to interact with other signal-transducing proteins and competently generate mitogenic signal [22]. The observed reduction in the expression of the bovine VEGFA-121 and VEGFR-1 mRNA in this study at Days 15 and 18, respectively, could in part be a resultant of higher P4 concentrations in pregnant heifers at Days 15 and 18 than in the cycling heifers. In gilts, P4 likewise inhibited VEGFA and its receptor expression in the intercaruncular endometrium [21]. In the present study, we further observed an additional effect of E2 on P4 on VEGFR-1 mRNA expression. Hayashi et al. [23] suggested that the bovine endometrium is highly sensitive to peripheral E2 and might thus favour the mRNA expression of VEGF-family members during the estrous phase. As E2 levels decline post estrus, we found coincident lower levels of VEGF. Additionally, Tasaki et al. [24] indicated that the bovine endometrial VEGFR-1 mRNA expression is highest during estrus and the follicular period. A high sensitivity of the endometrium to hormones is further evidenced by a positive response to the hormone-like compounds such as prostaglandin E_2_, which is reported to upregulate VEGFA mRNA expression in the endometrium in bovine [25]. It is therefore justifiable that though we could only found numerical differences in E2 concentrations between the nonpregnant and pregnant heifers at Day 18, E2 impacting on the VEGFR-1 mRNA expression may be possible.

The lack of significant differences between Days 15 and 18 conceptuses for the ligands and the receptors indicates a potential absence of differential autocrine signaling in the conceptuses via VEGFA-VEGFR system from Days 15 to 18. Other proangiogenic growth factors such as the fibroblast growth factors may be comparatively critical between Days 15 and 18. Fibroblast growth factors were reported to induce the pregnancy recognition signal interferon-tau mRNA expression in vitro [26]. Interferon-tau secretion increases from Days 15 to 18 [25]. Nevertheless, the underlying mechanism with respect to the synergy between the proangiogenic factors and interferon-tau is yet to be fully investigated. Interestingly, we noted that the abundance of VEGFA mRNA in the intercaruncular endometrium was either comparable to (VEGFA-121) or slightly higher (VEGFA-165 and VEGFA-189) than the conceptus mRNA transcripts. Embryonic VEGFR-1 and -2 displayed a much lower mRNA expression than the endometrial expression. We therefore propose that the VEGFA of conceptus origin might affect the VEGF receptors expressed in the endometrium during the preimplantation phase. This might be necessary for proper interaction between the endometrium and the conceptus prior to implantation in bovine. In humans, it is known that the conceptus-derived VEGFA induces vasodilatation and angiogenesis [27]. Additionally, the expression of VEGFA and VEGFR-1 in the human conceptuses further supports the likely regulatory role of VEGFA on conceptus proliferation, invasion and associated metabolic activities during implantation [27].

Our consistent observation of high VEGFA-165 expression in the endometrium and the conceptuses across our three experiments confirms the previous reports that VEGFA-165 is regarded as the prototypical isoform among the VEGFA isoforms [6,7,8]. The VEGFA-165 has a higher binding capactiy to its receptors, heparin and components of the extracellular matrix [6,7]. Peach et al. [6] performed computational modelling and indicated order of the mRNA abundance is VEGFA-165 > VEGFA-189 > VEGFA-121 in total tissue, extracellular matrix and plasma with VEGFA concentrations of less than 30 pM. The expression of VEGFA-165 also induced the highest levels of phosphorylation via VEGFR-2 [6]. In our study, we observed a significant change in VEGFR-1 mRNA expression, which is considered a decoy receptor to VEGFR-2 due to its decreased tyrosine kinase [6].

The VEGFA protein was localised in the intercaruncular luminal and glandular epithelium as well as in the tunica muscularis of blood vessels of the cycling and pregnant heifers without differing in intensity over time. We therefore tested the hypothesis that caruncular endometrial areas displayed a higher VEGFA abundance in preparation for implantation than intercaruncular endometrium. The endometrial glands that synthesise the uterine fluid are largely present in the intercaruncular endometrium, which has been shown to be critical for the development of the conceptus [15]. The caruncular endometrium, on the other hand, fuses with embryo cotyledons to form placentome during pregnancy [15]. We also observed significantly higher VEGFA mRNA expression and a higher numerical difference in protein abundance at Day 15 in the caruncular endometrium area that forms part of the embryo-maternal interface. As such, we further affirmed that during the preimplantation period in cattle, there may be a need for timely physiological reduction in the intercaruncular endometrial tissue VEGFA expression in favour of caruncular endometrial tissue to facilitate a gradient towards the implantation sites.

We observed a significantly higher protein abundance in the intercaruncular endometrium of heifers carrying SCNT than IVF-produced conceptuses at Day 18. The placental failure in bovine cloned pregnancies has been shown to be a consequence of disrupted conceptus–endometrium signalling developing during the preimplantation period [16,18]. Mess et al. [10] suggested a compensatory growth in response to the abnormal vascular development occurring in early pregnancy stages following an observation of overexpression of the VEGFA in IVF and in SCNT pregnancies [10]. Similar to previous results [10], we observed a significantly higher VEGFA mRNA expression in the IVF than SCNT. However, clones show substantial individual differences in differential gene expression [10]. The contrasting results we observed regarding the protein and mRNA expression in the cloned pregnancies could be due to differences in the posttranscriptional and translational regulation and protein degradation [28]. There is a higher rate of protein translation than mRNA transcription [28]. Additionally, the half-life of diverse proteins varies from minutes to days while the degradation rate of mRNA is within hours [28]. Assisted reproductive technologies, in particular SCNT, have been shown to result in restricted fetal growth in the earlier stages of pregnancy and later an increased growth in placental tissues until term in humans [10,29]. It appears that in bovine, the accelerated growth may originate from abnormalities occurring as early as Day 18 post insemination. The abnormality in cloned pregnancies to some extent could therefore relate to disturbed vascular development during the preimplantation period, originating from failure in proper embryo–mother communication.

## 4. Materials and Methods

All experiments were performed with permission from the local veterinary authorities and in accordance with the acceptable standards regarding treatment and handling of animals. The animal experiment was conducted in Freising, Germany and approved by the District Government of Upper Bavaria and was in accordance with the accepted standards of humane animal care in Germany.

### 4.1. Study A: Cycling versus Pregnant Heifers

#### 4.1.1. Animals and Collection of Samples

Cyclic Simmental heifers of an average age of 23 months were synchronised and treated with Estrumate (cloprostenol sodium) (500 mg i.m., Essex Tierarznei, Munich, Germany) at diestrus as previously described [30]. Briefly, after estrous detection, the animals were randomly assigned to their treatment groups. The pregnant groups (*n* = 5 per group) were inseminated with cryo-preserved semen while the nonpregnant control groups (*n* = 5 to 8 per group) were inseminated with the supernatant of centrifuged semen from the bull of the same breed. Animals were slaughtered at Days 12, 15 and 18 post insemination (insemination day = Day 0). Blood samples were obtained before slaughter to determine serum progesterone (P4) and estradiol-17β (E2) levels. Within twenty minutes after slaughter, the uterus was removed and flushed with phosphate buffered saline (PBS, pH 7.4) for the recovery of conceptuses. The uterus was longitudinally opened to sample endometrial tissue. Pregnancy was confirmed by the presence of a conceptus in the uterine lumen. For gene expression analysis, endometrial tissue samples ipsilateral to the ovary bearing the corpus luteum were sampled from the lamina propria using a scalpel, as described previously [30]. Tissue sample aliquots for gene expression analysis were immediately transferred into tubes containing RNA later and managed as per manufacturer’s instructions (Ambion, Huntington, UK). For determination of endometrial VEGFA protein levels, tissue sample aliquots were snap-frozen in liquid nitrogen. All samples were stored at −80 °C until further analysis. For localisation of VEGFA in the endometrium, samples were fixed in Bouin’s solution, washed with ethanol in series and embedded in paraffin [31].

#### 4.1.2. Analyses of P4 and E2 Concentration in Serum

Levels of progesterone (ng/mL) and estradiol-17β (pg/mL) in the serum were quantified using an enzyme-linked immunosorbent assay, as described by Prakash et al. [32]. Briefly, 0.5 mL plasma were mixed with 5 mL of *tert*-butyl methyl ether mixed with petroleum ether (30/70 v/v; AppliChem, Darmstadt, Germany) and incubated for two hours at room temperature under constant agitation. The samples were then stored at −60 °C for at least one hour. Thereafter, the supernatant was decanted and evaporated to dryness using a speed vac (Genevac EZ-2, StepBio, Bologna, Italy). Thereafter, the dried residues were redissolved in 0.4 mL of assay buffer. The plasma progesterone concentration was quantified using an enzyme-linked immunosorbent assay. The detection limit of the assay was 0.4 ng/mL. The intra- and inter-assay coefficients of variation were less or equal to 10% and 12%, in that order.

#### 4.1.3. Gene Expression Analysis of VEGFA Isoforms and VEGF Receptors in Endometrial and Conceptus Tissues

Total RNA isolation and gene expression analysis were conducted as previously described [30,33]. The total RNA from endometrial and conceptus tissue samples was isolated using TRIzol reagent (Invitrogen Corporation, Carlsbad, CA, USA). The quality of the RNA was assessed using Agilent 2100 Bioanalyzer (Agilent Technology, Palo Alto, CA, USA). The RNA integrity numbers (RIN) of the samples were at least 7. The RNA amount was spectrophotometrically determined at 260 nm by a Nanodrop 1000 ND-1000 (peqLab Biotechnologie GmbH). GoScript™ Reverse Transcription System kit was used to synthesise complementary DNA (cDNA) as per manufactures instructions (Promega, Switzerland) and the quantitative PCR reactions were carried out using the LightCycler DNA Master SYBR Green I protocol (Roche Diagnostics). The gene expression of the target genes was normalised against the reference gene polyubiquitin (UBQ3) to generate a relative RNA expression (ΔCq). To avoid negative values while permitting an evaluation of a relative comparison between two genes, the resulting ΔCq value was subtracted from an arbitrary value of 20. Thus, high ΔCq values represent high transcript abundances. The primer pairs for all the genes analysed are presented in Table 2.

#### 4.1.4. VEGFA Protein Concentration in Endometrial Homogenates

Endometrial samples were homogenised in a MagNa Lyser instrument (Roche Applied Science, Mannheim, Germany) and centrifuged through a NucleoSpin Filter L (Macherey-Nagel GmbH & Co KG, Düren, Germany). The protein concentrations of endometrial homogenates were determined using a bicinchoninic acid standard protocol [31]. Endometrial VEGFA protein concentrations were determined using the commercially available DuoSet Development VEGF ELISA DY293b (R&D Systems Inc., Minneapolis, MN, USA) according to the manufacturer’s instructions. Results are presented as means pg/mg total protein ± standard error of the mean (SEM).

#### 4.1.5. Immunolocalisation of VEGFA in the Bovine Endometrium

Deparaffinised and rehydrated uterine tissue sections (4 µm) were incubated for 15 min in methanol containing 1% H_2_O_2_ to quench endogenous peroxidase. For heat-induced antigen retrieval, tissue sections were boiled in 0.1 M citrate buffer for 15 min followed by a cooling step for 30 min at room temperature. Inside a humidified chamber, nonspecific binding was blocked with 10% goat serum (Dako, Glostrup, Denmark) and washed in PBS with 0.05% Tween20 (PBST) for 30 min. The primary polyclonal VEGFA IgG antibody (kindly provided by Prof. D. Schams, Weihenstephan, Germany) directed against each of the bovine VEGFA isoforms (Clone Ka21) was added in a 1:50 dilution on the slides and incubated overnight at 4 °C. Upon washing in PBST, the secondary antirabbit horseradish-conjugated IgG (Sigma-Aldrich, Steinheim, Germany) was added to the slide in a concentration of 2.5 µg/mL for 30 min. Antibody binding was detected by incubating the slides in PBST containing 3,3′-diaminobenzidintetrahydrochloride (DAB) and 0.1% H_2_O_2_ for 2 to 10 min. Cell nuclei were stained using Mayer’s Haemalaun (Carl Roth GmbH, Karlsruhe, Germany) prior to rehydratation and mounting. Microphotographs were taken by using the Zeiss Axioskop 2 and AxioVision 3.1 Software (Zeiss, Jena, Germany).

### 4.2. Study B: Caruncular versus Intercaruncular Endometrium in Pregnant Heifers

Cyclic Angus heifers of average age 19 months were supplemented with 450 g of rumen-protected sunflower oil, rich in omega-6 fatty acids, which affects follicular development and oocyte competency [33], for a period of 8 weeks (*n* = 15). The heifers were cycle synchronised, inseminated with cryo-preserved semen and slaughtered at Day 15 of pregnancy, described in detail by Giller et al. [33]. The tissue sampling was similar to that described for study A. Intercaruncular and caruncular endometrium was sampled. The intercaruncular and caruncular endometrium areas have both structural and functional differences regarding pregancy establishment [15]. Samples from pregnant heifers with a conceptus of 6mm or less were used for further analysis (*n* = 12). Gene expression analysis of the VEGFA isoforms and the VEGFR-1 and VEGFR-2 was similar to that described for study A. The total RNA was isolated using RNA Mini Kit (Qiagen, Hilden, Germany) according to the manufacturer’s instructions, and the RIN values were at least 5.5. A commercial Bovine VEGF-A ELISA kit (Merck, Switzerland) was used to quantify the VEGFA protein concentration in the caruncular and intercaruncular endometrium homogenates as per manufacturer’s instructions.

### 4.3. Study C: SCNT versus IVF Pregnancies

In vitro fertilisation (IVF) and SCNT procedures were carried out as previously described [16]. Two IVF or two SCNT blastocysts of grade 1 were transferred in estrus-synchronised Simmental recipient heifer (*n* = 8 per group) at Day 7 of the estrous cycle and slaughtered at Day 18 of pregnancy. Uteri were flushed with 100 mL of pre-warmed PBS for the recovery of embryos and endometrial tissue sampling. The tissue sampling and the SCNT and IVF intercaruncular endometrium and conceptuses gene expression analysis was performed as described for study A. The normalised ΔCq value was subtracted from an arbitrary value of 24. The protein abundance analyses were performed as described for study B.

### 4.4. Statistical Analysis

All the statistical tests were performed using IBM SPSS Statistics for Windows, Version 24.0 (Armonk, NY: IBM Corp.), and the data was normally distributed. To evaluate differences in endometrial gene expression, a linear mixed model was used and the variables in the model were day of the estrous cycle (Day), pregnancy status [pregnant vs. nonpregnant (Status)] and the interaction between the day of the cycle and pregnancy status (Day × Status). In study A, the concentration of P4 and E2 at slaughter were included as covariates. Bonferroni test was used in posthoc analysis. A *t*-test was used to determine the significant differences in conceptus gene expression between Days 15 and 18, and the gene expression and VEGFA protein abundances between IVF and SCNT pregnancies. Graphs were plotted using GraphPad Prism for Windows, version 7.03 (GraphPad Software, La Jolla, CA, USA). The results were considered significantly different when *p* < 0.05. We further calculated the fold numerical difference between the endometrium and conceptus normalised ΔCq in an attempt to understand the embryo–mother interaction.

## 5. Conclusions

Taken together, we have bridged the knowledge gap between already known critical functions of VEGFA and VEGF receptors during folliculogenesis [14], embryogenesis until Day 8 [12] and maintenance of pregnancy from early implantation period, between Days 18 and 24, to term [1] in bovine. We have reported novel findings regarding the expression of VEGFA isoforms and VEGF membrane receptors during the preimplantation period in bovine. We speculate about a contribution of VEGFA-121 during the initiation process of implantation starting Day 15 via VEGFR-1 receptor specifically in the caruncular endometrium areas that are the sites of implantation. The higher VEGFA protein abundance in the intercaruncular endometrium of heifers in response to SNCT embryos may indicate the placentation abnormalities frequently observed following SCNT.

## Figures and Tables

**Table 1:** Progesterone and estradiol-17β concentrations at slaughter in the serum of cyclic and early pregnant heifers after insemination (insemination = Day 0). Values presented as mean ± standard error of the mean (SEM). Probability value (*p*-value) for the respective pairs is given.

Day	Status	Animals	Progesterone [ng/mL]		Estradiol-17β [pg/mL]	
			Mean ± SEM	*p*-Value	Mean ± SEM	*p*-Value
Day 12	nonpregnant	6	8.40 ± 1.02	0.7	3.51 ± 0.61	0.7
	pregnant	5	7.83 ± 0.93		3.23 ± 0.56	
Day 15	nonpregnant	7	6.63 ± 0.86	0.04	1.92 ± 0.52	0.3
	pregnant	6	9.37 ± 0.93		2.81 ± 0.56	
Day 18	nonpregnant	8	6.80 ± 0.80	0.003	1.20 ± 0.48	0.2
	pregnant	5	10.91 ± 1.02		2.32 ± 0.61	

**Table 2:** Forward (for) and reverse (rev) primer sequences of the reference gene polyubiquitine, vascular endothelial growth factor A (VEGFA) isoforms and VEGF receptors (VEGFR-1 and VEGFR-2) for bovine used in quantitative reverse transcription-PCR.

Primer	Sequence		Fragment Length [bp]	Accession Number
Polyubiquitin	for	AGATCCAGGATAAGGAAGGCAT	198	NM_174133
	rev	GCTCCACCTCCAGGGTGAT		
VEGFA-121	for	CCGTCCCATTGAGACCCTG	208	AB455252
	rev	CGGCTTGTCACAATTTTTCTTGTC		
VEGFA-165	for	CCGTCCCATTGAGACCCTG	278	NM_174216
	rev	GCCCACAGGGATTTTCTTGC		
VEGFA-189	for	CCGTCCCATTGAGACCCTG	297	AB450824
	rev	TGCCCTTTGCCCTTTCCTC		
VEGFR-1	for	CACCAAGAGCGACGTGTG	351	X94263
	rev	AAGAAGTCCTCGGAGAAGGC		
VEGFR-2	for	AGACTGGTTCTGGCCCAAC	379	X94298
	rev	GAAGCCTTTCTGGCTGTC		

## References

[1] Pfarrer C.D., Ruziwa S.D., Winther H., Callesen H., Leiser R., Schams D., Dantzer V. (2006). Localization of vascular endothelial growth factor (VEGF) and its receptors VEGFR-1 and VEGFR-2 in Bovine placentomes from implantation until term. Placenta.

[2] Torry D.S., Leavenworth J., Chang M., Maheshwari V., Groesch K., Ball E.R., Torry R.J. (2007). Angiogenesis in implantation. J. Assist. Reprod. Genet..

[3] Mackenzie F., Ruhrberg C. (2012). Diverse roles for VEGF-A in the nervous system. Development.

[4] Iyer S., Acharya K.R. (2011). Tying the knot: The cystine signature and molecular-recognition processes of the vascular endothelial growth factor family of angiogenic cytokines. FEBS J..

[5] McFee R.M., Rozell T.G., Cupp A.S. (2012). The balance of proangiogenic and antiangiogenic VEGFA isoforms regulate follicle development. Cell Tissue Res..

[6] Peach C.J., Mignone V.W., Arruda M.A., Alcobia D.C., Hill S.J., Kilpatrick L.E., Woolard J. (2018). Molecular pharmacology of VEGF-A isoforms: binding and signalling at VEGFR2. Int. J. Mol. Sci..

[7] Bridgett S., Dellett M., Simpson D.A. (2017). RNA-Sequencing data supports the existence of novel VEGFA splicing events but not of VEGFAxxxb isoforms. Sci. Rep..

[8] Sarabipour S., Mac-Gabhann F. (2018). VEGF-A121a binding to Neuropilins—A concept revisited. Cell. Adh. Migr..

[9] Karkkainen M.J., Petrova T.V. (2000). Vascular endothelial growth factor receptors in the regulation of angiogenesis and lymphangiogenesis. Oncogene.

[10] Mess A.M., Carreira A.C.O., Marinovic D.-O.C., Fratini P., Favaron P.O., Barreto R.-D.-S.N., Pfarrer C., Meirelles F.V. (2017). Vascularization and VEGF expression altered in bovine yolk sacs from IVF and NT technologies. Theriogenology.

[11] Luo H., Kimura K., Aoki M., Hirako M. (2002). Vascular endothelial growth factor (VEGF) promotes the early development of bovine embryo in the presence of cumulus cells. J. Vet. Med. Sci..

[12] Luo H., Kimura K., Aoki M., Hirako M. (2002). Effect of vascular endothelial growth factor on maturation, fertilization and developmental competence of bovine oocytes. J. Vet. Med. Sci..

[13] Anchordoquy J.M., Anchordoquy J.P., Testa J.A., Sirini M.Á., Furnus C.C. (2015). Influence of vascular endothelial growth factor and Cysteamine on in vitro bovine oocyte maturation and subsequent embryo development. Cell Biol. Int..

[14] Ortega-Serrano P.V., Guzmán A., Hernández–Coronado C.G., Castillo-Juárez H., Rosales-Torres A.M. (2016). Reduction in the mRNA expression of sVEGFR1 and sVEGFR2 is associated with the selection of dominant follicle in cows. Reprod. Domest. Anim..

[15] Mansouri-Attia N., Aubert J., Reinaud P., Giraud-Delville C., Taghouti G., Galio L., Everts R.E., Degrelle S., Richard C., Hue I. (2009). Gene expression profiles of bovine caruncular and intercaruncular endometrium at implantation. Physiol. Genom..

[16] Bauersachs S., Ulbrich S.E., Zakhartchenko V., Minten M., Reichenbach M., Reichenbach H.-D., Blum H., Spencer T.E., Wolf E. (2009). The endometrium responds differently to cloned versus fertilized embryos. Proc. Natl. Acad. Sci. USA.

[17] Panarace M., Agüero J.I., Garrote M., Jauregui G., Segovia A., Cané L., Gutiérrez J., Marfil M., Rigali F., Pugliese M. (2007). How healthy are clones and their progeny: 5 years of field experience. Theriogenology.

[18] Mansouri-Attia N., Sandra O., Aubert J., Degrelle S., Everts R.E., Giraud-Delville C., Heyman Y., Galio L., Hue I., Yang X. (2009). Endometrium as an early sensor of in vitro embryo manipulation technologies. Proc. Natl. Acad. Sci. USA.

[19] Azimi-Nezhad M. (2014). Vascular endothelial growth factor from embryonic status to cardiovascular pathology. Rep. Biochem. Mol. Biol..

[20] Cheung C.Y. (1997). Vascular endothelial growth factor: Possible role in fetal development and placental function. J. Obstet. Gynaecol. Res..

[21] Kaczmarek M.M., Waclawik A., Blitek A., Kowalczyk A.E., Schams D., Ziecik A.J. (2008). Expression of the vascular endothelial growth factor-receptor system in the porcine endometrium throughout the estrous cycle and early pregnancy. Mol. Reprod. Dev..

[22] Ferrara N., Gerber H.-P., LeCouter J. (2003). The biology of VEGF and its receptors. Nat. Med..

[23] Hayashi K.-G., Hosoe M., Fujii S., Kanahara H., Sakumoto R. (2019). Temporal expression and localization of vascular endothelial growth factor family members in the bovine uterus during peri-implantation period. Theriogenology.

[24] Tasaki Y., Nishimura R., Shibaya M., Lee H.Y., Acosta T.J., Okuda K. (2010). Expression of VEGF and its receptors in the bovine endometrium throughout the estrous cycle: Effects of VEGF on prostaglandin production in endometrial cells. J. Reprod. Dev..

[25] Zhang S., Liu B., Mao W., Li Q., Fu C., Zhang N., Zhang Y., Gao L., Shen Y., Cao J. (2017). The effect of prostaglandin E2 receptor (PTGER2) activation on growth factor expression and cell proliferation in bovine endometrial explants. Prostaglandins Leukot. Essent. Fat. Acid..

[26] Cooke N.T.F., Pennington A.K., Yang Q., Ealy D.A. (2009). Several fibroblast growth factors are expressed during pre-attachment bovine conceptus development and regulate interferon-tau expression from trophectoderm. Reproduction.

[27] Boudjenah R., Molina-Gomes D., Wainer R., de Mazancourt P., Selva J., Vialard F. (2012). The vascular endothelial growth factor (VEGF) +405 G/C polymorphism and its relationship with recurrent implantation failure in women in an IVF programme with ICSI. J. Assist. Reprod. Genet..

[28] Vogel C., Marcotte E.M. (2012). Insights into the regulation of protein abundance from proteomic and transcriptomic analyses. Nat. Rev. Genet..

[29] Bloise E., Feuer S.K., Rinaudo P.F. (2014). Comparative intrauterine development and placental function of ART concepti: Implications for human reproductive medicine and animal breeding. Hum. Reprod..

[30] Ulbrich S.E., Schulke K., Groebner A.E., Reichenbach H.D., Angioni C., Geisslinger G., Meyer H.H. (2009). Quantitative characterization of prostaglandins in the uterus of early pregnant cattle. Reproduction.

[31] Groebner A.E., Schulke K., Unterseer S., Reichenbach H.D., Reichenbach M., Büttner M., Wolf E., Meyer H.H.D., Ulbrich S.E. (2010). Enhanced proapoptotic gene expression of XAF1, CASP8 and TNFSF10 in the bovine endometrium during early pregnancy is not correlated with augmented apoptosis. Placenta.

[32] Prakash B.S., Meyer H.H., Schallenberger E., van de Weil D.F. (1987). Development of a sensitive enzymeimmunoassay (EIA) for progesterone determination in unextracted bovine plasma using the second antibody technique. J. Steroid Biochem..

[33] Giller K., Drews B., Berard J., Kienberger H., Schmicke M., Frank J., Spanier B., Daniel H., Geisslinger G., Ulbrich S.E. (2018). Bovine embryo elongation is altered due to maternal fatty acid supplementation. Biol. Reprod..

